# CIT kinase phosphorylation as significant regulatory node for cellular checkpoints

**DOI:** 10.3389/fbinf.2025.1734030

**Published:** 2026-01-12

**Authors:** Jaytha Thomas, Fathimathul Lubaba, Mukhtar Ahmed, Althaf Mahin, Levin John, Athira Perunelly Gopalakrishnan, Suhail Subair, Prathik Basthikoppa Shivamurthy, Rajesh Raju, Sowmya Soman

**Affiliations:** 1 Centre for Integrative Omics Data Science (CIODS), Yenepoya (Deemed to be University), Mangalore, Karnataka, India; 2 Department of Zoology, College of Science, King Saud University, Riyadh, Saudi Arabia; 3 Institute for Regeneration and Repair, University of Edinburgh, Edinburgh, United Kingdom

**Keywords:** autophosphorylation, cell cycle regulation, cellular checkpoint, CIT, citron, DNA repair, phosphoproteomics

## Abstract

**Introduction:**

Citron Rho-interacting serine/threonine kinase (CIT) is a major cytosolic protein kinase essential for midbody organisation, abscission, and cytokinesis. Dysregulation and mutations in CIT are associated with multiple cancers and neurodevelopmental disorders, including microcephaly. Although global phosphoproteomic studies have identified more than 50 phosphosites in CIT, their functional relevance and the kinases regulating them remain largely unexplored.

**Methods:**

To systematically investigate the phosphoregulation of CIT, we curated and integrated global phosphoproteomic datasets, along with their associated experimental conditions, to comprehensively catalogue phosphorylation events reported for CIT. To assess the functional significance of CIT, we examined proteins that were differentially co-regulated with its predominant phosphosite.

**Results:**

Serine 440 (S440), located outside the kinase domain (representing over 55% of CIT-associated phospho-signalling events across 100 experimental conditions, including Enterovirus A71 infection, metformin, and interleukin-33), was identified as its predominant phosphosite. Motif analysis revealed the presence of a D(S/T)P/P(S/T)D motif recognised by the CIT kinase domain, suggesting S440 as a predicted autophosphorylation site. Co-phosphoregulation analysis identified 136 interacting proteins and 82 predicted substrates that were positively co-regulated with CIT_S440. The resulting phospho-regulatory network comprised essential cell cycle and DNA repair regulators, including MDC1 and TRIP12. Significantly, over 120 co-regulated phosphosites were functionally linked to DNA repair and cell cycle regulation. Aberrant phosphorylation of CIT_S440 observed across cancers of the breast, colon, and bladder suggests CIT_S440 as a potential onco-phosphosite critically involved in cellular checkpoint signalling.

**Discussion:**

These findings suggest that CIT_S440 functions as a promising therapeutic target, and the phosphosite-centric regulatory network derived in this study could serve as a platform to evaluate its phosphosite-specific therapeutic interventions.

## 1 Introduction

Citron Rho-interacting serine/threonine kinase (CIT) is a multifunctional member of the AGC kinase family with established roles in cytokinesis, central nervous system (CNS) development, and cancer progression (Di Cunto et al., 1998; Meng et al., 2019; Bianchi et al., 2020). CIT, located on chromosome 12q24.23, encodes a 2027 amino acid-long protein with an approximate molecular weight of 230 kDa (Di Cunto et al., 1998; Bianchi et al., 2020). CIT is also known by multiple synonyms, including CIT-K (McKenzie et al., 2016), CRIK (Di Cunto et al., 1998), MCPH17 (Pallavicini et al., 2021) and STK21(Yao et al., 2019). Structurally, CIT comprises an N-terminal kinase domain, two central coiled-coil domains (CC1 and CC2) containing a Rho/Rac-binding domain (RBD) and a cysteine-rich (C1) motif adjacent to a pleckstrin homology (PH) domain, and a C-terminal Citron-Nik1 homology (CNH) responsible for mediating multiple protein-protein interactions (D’Avino, 2017). CIT exists in two isoforms, CIT-K with a kinase domain and CIT-N lacking kinase-domain (D’Avino, 2017). CIT exhibits dynamic subcellular localization during cell division, accumulating at the spindle midzone during anaphase, relocating to the cleavage furrow in telophase, and concentrating at the midbody during cytokinesis, thereby ensuring proper daughter cell separation (D’Avino, 2017; LoTurco et al., 2003).

Beyond its established role in cytokinesis, CIT contributes to diverse processes such as CNS development, maintenance of genome stability (Bianchi et al., 2020), regulation of abscission (Gai et al., 2011), chromatin remodeling (Rawat et al., 2023), and signaling pathways such as the Hippo signaling pathway, which regulates cell growth and division, and the homologous recombination pathway, where CIT facilitates RAD51 accumulation at DNA double-strand breaks (Rawat et al., 2023; Tran et al., 2019; Bianchi et al., 2017). CIT-kinase has also been linked to various diseases due to alterations in its expression status and sequence variations. Recent studies elucidated mutated CIT (Asp221Ter, Asp230Val) as a potential effector of the DNA repair pathway leading to microcephaly (Ribeiro et al., 2023). Its overexpression is observed in various cancers, including bladder, breast, and colon cancer (Shou et al., 2020; Meng et al., 2019; Wu et al., 2017). However, a report from McKenzie et al. suggests that CIT overexpression alone is not sufficient to induce oncogenicity (McKenzie and D’Avino, 2016).

As a kinase, activation of CIT and interactions with proteins are likely regulated by post-translational modifications. Phosphorylation, a pivotal modification, can govern protein function, localization, and signalling (Raju et al., 2011; Krug et al., 2019; Gopalakrishnan et al., 2025). CIT can autophosphorylate (Di Cunto et al., 1998) and is also phosphorylated by other kinases, such as SRC, and enhances the association of active RhoA (Jungas et al., 2016) through Ephrin/Eph receptor signalling to modulate abscission *via* tyrosine phosphorylation (Jungas et al., 2016; D’Avino, 2017). Moreover, CIT is shown to phosphorylate substrates such as the Regulatory Myosin Light Chain (RMLC) to facilitate cytokinesis (Yamashiro et al., 2003).

While CIT has been studied at the protein level, its post-translational modifications, particularly phosphorylation, are considerably unexplored. This study examines the global phosphoregulatory network of the CIT kinase by systematically analysing the mass-spectrometry-based phosphoproteomics datasets to investigate the phosphosite-specific regulation of CIT and the broader network of co-regulated proteins, its downstream substrates, and the binary interactors associated with its predominant phosphorylation sites.

## 2 Materials and methods

### 2.1 Curation and assembly of CIT phosphopeptides from global phosphoproteomics datasets

To screen the global phosphoproteomics datasets in which CIT phosphopeptides are detected, we conducted a PubMed query using the terms “phosphoproteomics” OR “phosphoproteome”. NOT “Plant”, NOT “Review”, Our analysis mainly focused on published datasets that provided a global analysis of the human cellular and tissue-level phosphoproteome, and we manually curated the high-throughput datasets that contain the CIT phosphopeptides. Class-1 phosphosites (highest confidence in localization) were captured based on criteria such as localization probability (≥75%) and/or ambiguity score (A-score >13). These datasets were further classified into qualitative profile datasets, which contain all the detected phosphopeptides (test conditions and controls are treated as independent phosphoproteome datasets), and quantitative differential profile datasets, which consist of differentially regulated phosphopeptides (comparison of specific biological or experimental conditions to their respective controls). In differential profile datasets, we applied the study-centric statistical parameters (p-value <0.05), and Class-1 phosphosites were classified as either upregulated (≥1.3-fold change) or downregulated (≤0.76-fold change). These datasets were further categorized based on their enrichment analysis techniques (STY/ST/Y phosphosites). Using our in-house mapping tool, each protein accessions were mapped to its corresponding UniProt (downloaded on May 13, 2023) (UniProt Consortium, 2023) accession versions and the proteins to their corresponding gene symbol (Seal et al., 2023) phosphosites were further mapped against their corresponding UniProt FAST to warrant their consistent mapping. Following a standard annotation strategy, each dataset was annotated along with its biological and experimental conditions for effective classification, considering the previous studies (Mahin et al., 2025; Priyanka et al., 2024).

### 2.2 Identification of phosphorylation sites prominently altered in CIT

To identify the predominant sites of CIT, the Class-1 phosphosites assembled from the human cellular phosphoproteome datasets were subjected to further analysis. We computed and ranked the total number of qualitative profile datasets where each CIT phosphosite was identified and quantitative differential profile datasets where the CIT phosphosite was shown to be differently regulated. Arbitrarily, the phosphosites detected in over 50% of qualitative profile and quantitative differential profile datasets were considered as the predominant phosphosites of CIT kinase (Lalu et al., 2025; Raghu et al., 2025). The phosphosites identified through specific phospho-antibodies or mutation-based approaches, which are not frequently detected or classified as class-1 sites in phosphoproteomics datasets, were not considered as predominant sites (Sanjeev et al., 2024; John et al., 2024).

### 2.3 Co-occurrence analysis of CIT phosphosites

Co-occurring protein phosphorylation events have been reported to be functionally associated (Li et al., 2017). To investigate the mutual associations of phosphorylation sites within CIT, we conducted a co-occurrence analysis that specifically examined the co-differential regulation pattern of phosphosite pairs within CIT. Incorporating differential datasets, multiple phosphosites within CIT co-detected under same experimental conditions were analyzed. For each pair, we separately calculated the frequencies and further assessed their positive and negative co-regulation patterns. The differential co-regulation frequency of the phosphosite pair is listed in Supplementary Table S1A.

### 2.4 Filtering the phosphosites in other proteins (PsOPs) that are co-detected/regulated with CIT_S440, the predominant site

To determine the phosphosites in other proteins (PsOPs) that show either positive or negative co-regulation in expression with S440 of CIT, the differential datasets corresponding to each phosphosite were categorised separately. Due to the large number of datasets spanning the various experimental conditions, biological systems, and different analysis platforms, re-analysis of raw datasets was not possible. The phosphosites that follow the criteria, such as localization probability (≥75%) and ambiguity score (A-score >13), were considered for further analysis. The quantitative differential profile datasets were further categorised based on the differential regulation of CIT_S440 (c) compared to the co-differentially regulated PsOPs (o) that showed co-differential regulation with CIT_S440. Datasets with upregulated CIT_S440 are represented as Uc, and those with downregulated CIT_S440 are represented as Dc. Additionally, PsOPs (o), which were identified to be upregulated and downregulated when CIT_S440 is upregulated (UcUo and UcDo), and those which were upregulated and downregulated when S440 in CIT is downregulated (DcUo and DcDo) were extracted from each category.

Further, we computed the differential datasets in which each of the PsOPs formed the relationship with CIT_S440 under the UcUo, DcDo, UcDo, and DcUo categories. The PsOPs that fall in the UcUo and DcDo categories and those in the UcDo and DcUo categories co-regulate positively and negatively with the CIT_S440 expression, respectively. For the CIT_S440 predominant site, PsOPs were regrouped into UcUoDcDo (positive, also called UUDD) and UcDoDcUo (negative, also called UDDU) categories across their respective datasets.

PsOPs with a p-value <0.05 in the UUDD and UDDU categories for the respective CIT predominant phosphosite were considered significant and selected for further analysis. The differential Fisher’s Exact Test (FET) score of the CIT predominant phosphosite and PsOPs pair was calculated to assess the likelihood of co-regulation patterns without considering the potential bias-inducing factors, including the large number of phosphosites reported in one or two studies or the over-representation of multi-temporal datasets for a single type of stimulus.

Fisher’s Exact Test (FET) was performed by constructing a contingency table for the predominant phosphosite identified in CIT (CIT_S440) and co-regulated protein phosphosites. Based on this contingency table, we have calculated FET p-value using the equation as follows; Fisher’s exact test (FET): equation

$$\Sigma p=\frac{\left(a+b\right)!\left(c+d\right)!\left(a+c\right)!\left(b+d\right)!}{n!}\Sigma \frac{1}{ai!\;bi!\;ci!\;di!}$$

In this equation, a= (n_0c0o)’ denotes the number of experimental conditions in which neither site was detected, b= (n_Uc0+n_Dc0+n_0Uo + n_0Do) denotes the number of experimental conditions in which only one of the two sites was detected as either up- or downregulated), while the other site was not detected, c= (n_UcDo + n_DcUo) denotes the number of experimental conditions in which both sites exhibit opposite co-regulation (negative co-regulation), and d= (n_UcUo + n_DcDo) denotes the number of experimental conditions in which both sites exhibit identical co-regulation (positive co-regulation). Based on these values, contingency tables were generated, and Fisher’s Exact Test (FET) was employed to evaluate the significantly coregulated PsOPs with CIT_S440 (p-value <0.05).

The phosphosite pairs with a differential FET p-value <0.05 were further filtered based on the ratios of Ʃ (nUcUo + nDcDo)/Ʃ (nUcDo + nDcUo) for positively co-regulated PsOPs and Ʃ (nUcDo + nDcUo)/Ʃ (nUcUo + nDcDo) for negatively co-regulated PsOPs. The PsOPs that satisfy the ratio representing 10% of the total frequency of the S440 predominant site of CIT were considered significant and selected for further analysis. Additionally, we applied criteria such as PsOPs co-regulation with predominant phosphosite based on at least two distinct studies (PubMed IDs) and a diversity of at least two different experimental conditions (experimental code count). Overall, the PsOPs that showed either a positive or negative co-regulation were considered significant PsOPs when they met the four criteria mentioned above. To obtain insights into the functions of these high-confidence PsOPs, gene enrichment analysis was performed using DAVID (The Database for Annotation, Visualisation, and Integrated Discovery) (Sherman et al., 2022). These high-confidence PsOPs were subsequently analysed to explore their associated protein-protein interactions and kinase-substrate relationships with the predominant phosphosite, S440 of CIT.

### 2.5 Extraction of known and predicted kinase-substrate association of CIT

We retrieved the experimentally validated substrates of CIT_S440 along with their specific phosphosites enlisted in PhosphositePlus (Hornbeck et al., 2015) (downloaded on 22.05.2023), Phospho.ELM 9.0 (downloaded on 24.05.2023) (Dinkel et al., 2011), and RegPhos 2.0 (downloaded on 24.05.2023) (Huang et al., 2014). The predicted kinases and substrates of CIT were identified using multiple tools, including NetworKIN (downloaded on 04.01.2023) (Linding et al., 2008) and AKID (downloaded on 24.05.2023) (Priyanka et al., 2024; Parca et al., 2019). Subsequently, all the substrates and kinases of CIT were identified by Johnson et al., based on synthetic peptide screening to evaluate substrate specificity within the kinome and using a 90-percentile cutoff (Johnson et al., 2023).

### 2.6 Extraction of protein and phosphosite-specific interactors of CIT

We extracted experimentally validated protein-protein interactors along with their associated substrates using various databases, HPRD (Keshava Prasad et al., 2009), BIND (Baderet al., 2003), BioGRID (Oughtred et al., 2021), ConsensusPathDb release 35 (downloaded on 22.05.2023) (Kamburov and Herwig, 2022), CORUM (downloaded on 03.03.2023) (Tsitsiridis et al., 2023), and RegPhos 2.0 (downloaded on 24.05.2023) (Huang et al., 2014; Priyanka et al., 2024).

### 2.7 Data visualization

We used the R/Bioconductor package trackViewer (10.18129/B9.bioc.track-Viewer) to construct lollipop plots. The cumulative distribution of phosphosites across the quantitative differential profiling dataset was visualised using the Python packages Matplotlib and Pandas. Cytoscape (Shannon et al., 2003) is used for the interaction map visualisation, and RAWGraph 2.0 (Mauri et al., 2017) is used to plot dendrograms.

## 3 Results

### 3.1 A compendium of phosphorylated sites in CIT from global phosphoproteomes

To investigate the role of CIT in cellular phosphoproteomes, we analysed 758 profiling and 192 differential datasets, which consist of Class-1 phosphosites in CIT phosphopeptides. Specifically, 52 phosphosites in CIT were identified in the profile datasets, and 25 were reported as differentially regulated in diverse differential datasets. Among the 52 phosphosites, two were located within the kinase domain (S140 and T307), two within the PH domain (S1474 and S1488), and two within the CNH domain (S1624 and Y1678). No known functions are currently associated with any of these 52 phosphosites of CIT. To gain insights into their potential functional roles, we evaluated their regulatory patterns across diverse conditions. The complete list of CIT phosphosites in profile and differential datasets is given in Supplementary Table S1(B, C).

### 3.2 CIT_S440 was majorly perturbed in differential phosphoproteomes

**FIGURE 1:**
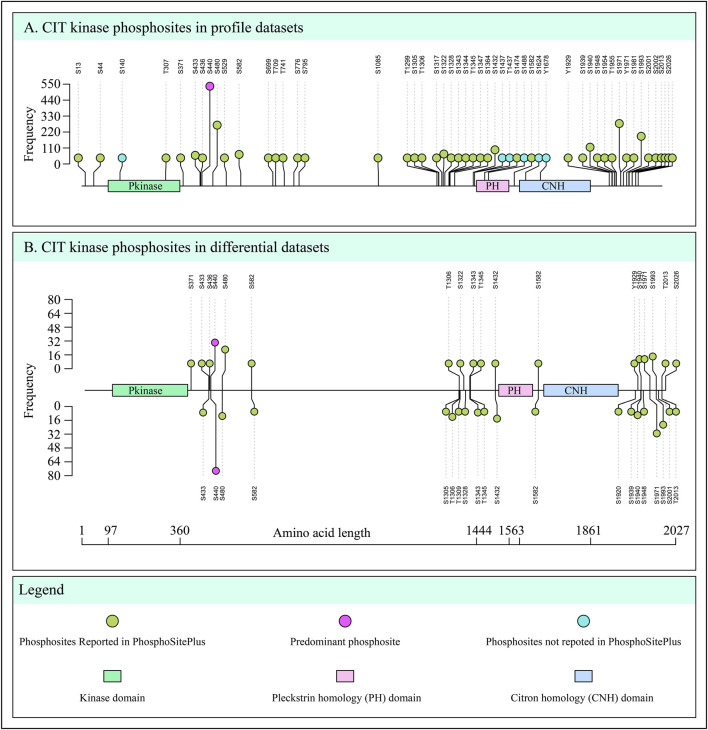
Lollipop plot illustrating the frequency of detection of phosphosites in CIT, with each node representing a specific phosphosite. **(A)** Class-1 phosphosites and their frequencies identified in 758 profile datasets. **(B)** Class-1 phosphosites and their frequencies were identified in 192 differential datasets. The upper frequency indicates the number of datasets in which the given phosphosites of CIT were upregulated, while the lower frequency indicates the number of datasets in which the CIT phosphosites were downregulated.

To detect the predominant site of CIT, we analyzed all Class-1 phosphosites, ranking them by their frequency of detection across 758 profiling datasets and 192 differential datasets. Out of the 52 phosphosites detected on the CIT phosphopeptides, several were detected with notable frequencies in the differential datasets. Specifically, we identified S440, S1971, 1993, S480, S1940, and S1432 as differentially regulated in 108, 46, 39, 37, 25, and 22 datasets, respectively. These six phosphosites were considered predominant due to their consistent detection across multiple datasets, indicating their significance in CIT phosphorylation. Among these, we primarily focused on Ser440, which was detected in 564 profiling datasets and 108 differential datasets, collectively accounting for approximately 55% of the total datasets analyzed. The high detection frequency of Ser440 underscores its status as the most significant CIT phosphosite, making it a primary target for further investigation. Figure 1 illustrates the phosphosites detected in the profile and differential datasets.

A recent study using LC-MS/MS-based *in vitro* kinase assay and *in vivo* phosphoproteome analysis by Sugiyama et al., 2019, suggested S440 as an autophosphorylation site (Sugiyamaet al., 2019). Even if the autophosphorylation of CIT is linked with Rho-Rac-binding (Di Cunto et al., 1998), it is still unclear whether any phospho-regulatory networks are connected with its function. Our primary focus is to explore and understand the co-regulatory phospho-centric patterns of CIT associated with its predominant phosphorylation site, Ser440.

### 3.3 Analysis of CIT phosphosite Co-occurrence patterns

Co-occurrence of phosphosites is often considered as a parameter to identify functional associations between phosphosites within a protein (Li et al., 2017). In the present study, we evaluated the co-occurrence patterns of predominant phosphosites on CIT across large-scale cellular phosphoproteome datasets to explore their regulatory relationships. Differential datasets showing differential expression of multiple CIT phosphosites within a single experimental condition were extracted for analysis. Phosphosite pairs that were simultaneously upregulated or downregulated were categorized as showing positive co-occurrence, whereas pairs exhibiting opposite regulation, one upregulated and the other downregulated, were considered to have negative co-occurrence.

For each phosphosite pair, the frequency of positive and negative co-occurrence was calculated across all differential phosphoproteomics datasets. Notably, phosphorylation at S1933 and S440 exhibited the most prominent co-occurrence pattern, being observed together in 15 differential datasets spanning 8 distinct experimental conditions. Among these, 13 datasets showed positive co-occurrence, with 8 datasets showing downregulation and 5 datasets showing upregulation of phosphorylation at these sites. The remaining two datasets demonstrated negative co-occurrence, suggesting an inverse regulatory relationship under IL-33 and Her2-positive/negative conditions. These patterns indicate a potential functional association between phosphorylation at S1933 and S440. Additional positive co-occurrence patterns were noted between S440 and S582 and between S1940 and S2026, each with a frequency of 6.

### 3.4 Phosphoproteins prominently co-regulated with CIT_S440 and their regulation in cancers

**FIGURE 2:**
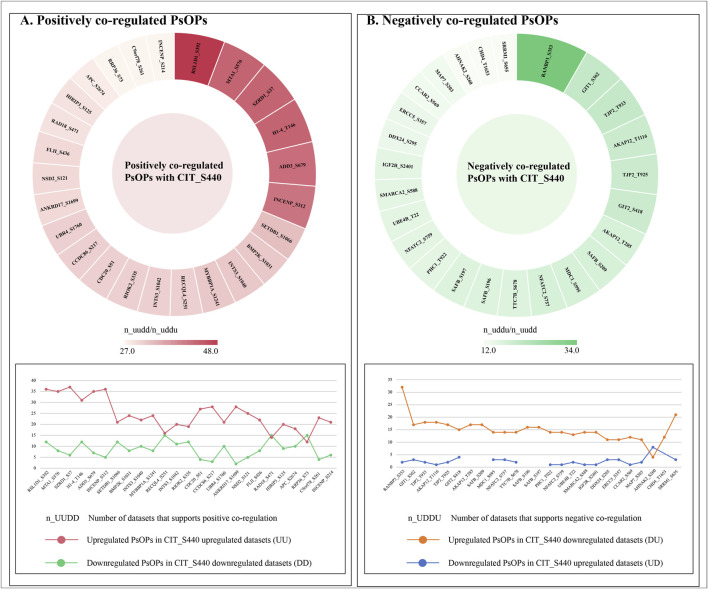
Top 25 high-confidence PsOPs co-regulation corresponding to Ser440 of CIT. Frequency value indicates the number of datasets in which the given phosphosite is detected.**(A)** Frequency distribution of the top 25 high-confidence PsOPs positively co-regulated (UUDD) with the predominant site Ser440 of CIT. **(B)** Frequency distribution of the top 25 high-confidence PsOPs negatively co-regulated (UDDU) with the predominant site Ser440 of CIT.

To interpret the impact of phosphorylation at Ser440 of CIT kinase, we conducted an extensive expression co-regulation analysis across the cellular differential phosphoproteome datasets. For evaluating the patterns, we employed the one-sided Fisher’s exact test (FET) to investigate the PsOPs that are either co-expressed or co-differentially regulated with the predominant site, Ser440 of CIT. To further improve the quality of the FET analysis, we employed stringent cut-off criteria based on the contingency table of differentially regulated proteins alongside the CIT_S440. We identified 793 and 43 high-confidence PsOPs that exhibit positive and negative co-regulation with CIT_S440, respectively. (Supplementary Table S1D,E) lists the high-confidence PsOPs that are positively and negatively co-regulated with Ser440 in CIT. RSL1D1_S392 (6.98E-11, 48, 6, 6) and MTA1_S576 (3.4E-06, 43, 8, 11) showed positive co-regulation, while RANBP3_S353 (3.73E-09, 34, 3, 4) and GIT1_S362 (0.001079, 20, 7, 10) showed negative co-regulation with CIT_S440. For each phosphosite, the values in parentheses represent the p-value, co-regulation frequency, PMID confidence, and experimental code confidence, respectively. Figure 2 shows the top 25 high-confidence PsOPs that are positively and negatively co-regulated with S440 of CIT. Co-regulated high-confidence PsOPs were used to identify the functions associated with CIT_S440.

**FIGURE 3:**
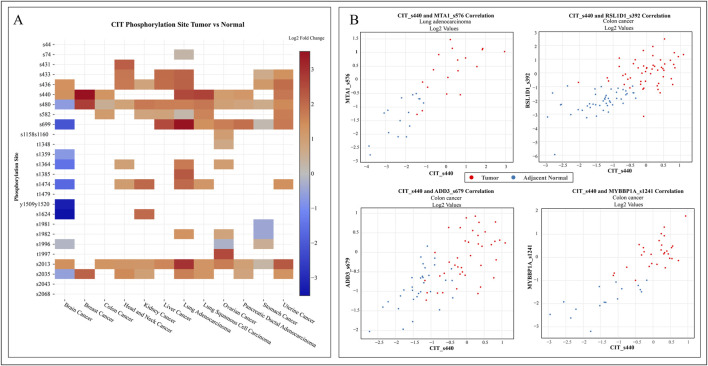
Expression profiles of phosphorylation sites in CIT across various cancer tissue samples sourced from the cProSite database. **(A)** Heatmap illustrating tissue-level expression patterns of phosphosites in CIT across multiple cancer types. **(B)** Scatter plot representing consistent expression correlation of CIT_S440 and co-regulated phosphosites in upstream kinases of tumor and adjacent normal samples.

Additionally, hyperphosphorylation of CIT_S440 is observed in multiple cancers, including colon, brain, breast, lung adenocarcinoma, lung squamous cell carcinoma, ovarian, pancreatic, and uterine cancers, as reported in cProSite (Wang et al., 2023). Furthermore, cProSite analysis revealed that, among the top positive co-regulations, phosphorylations at MYBBP1A_S1241, RSL1D1_S392, and ADD3_S679 were consistently co-regulated with CIT_S440 in colon cancer samples compared to adjacent normal samples. Similar correlation and hyperphosphorylation were also observed with MTA1_S576 and CIT_S440 in lung adenocarcinoma datasets (Figure 3). The quantitative data corresponding to the cProSite heatmap, including fold changes, p-values, and sample sizes, are provided in Supplementary Table S1K.

### 3.5 Inferring proteins and their phosphosites potentially associated with their interaction with CIT_S440

Protein-protein interactions mechanistically play a key role in regulating the functions of proteins. Several studies suggested that phosphorylation sites located near or within the protein-interacting interfaces can have either a positive or negative impact on the binding affinity and the stability of the proteins (Nishiet al., 2011). However, the lack of a complete crystal structure of CIT makes it difficult to interpret these molecular changes.

The phosphorylation of the CIT_S440 may offer insight into the interactive network of the CIT based on the co-differentially regulated proteins, if these proteins experience any conformational changes upon autophosphorylation. In a study by Capalbo et al., CIT interactors were investigated using affinity purification-mass spectrometry (AP-MS) (Capalbo et al., 2019). In the cellular phosphoproteomics datasets, we identified 136 binary interactors with 199 positively and 13 negatively co-regulated PsOPs with the predominant site of CIT (Supplementary Table S1F,G). The phospho-interactive network of CIT_S440 is based on the previously enlisted binary interactors. Among them, proteins such as SRRM2, INCENP, and MKI67 contain multiple phosphosites that are co-differentially regulated with CIT_S440, suggesting that these proteins may play an essential role in the regulation of CIT signalling pathways and potentially influence their functional effects through coordinated phosphorylation.

We also delved into the functions associated with these binary interactors. The serine/arginine repetitive matrix 2 (SRRM2) protein is crucial for organising nuclear speckles and regulating alternative splicing, which is involved in innate immunity and essential for maintaining protein function and cell homeostasis (Xu et al., 2022). Phosphoproteomics data identified that S440 of CIT differentially co-regulates with seven phosphosites of SRRM2 (S1010, S1219, S1621, S1732, S250, S914, and Y2693). The Mediator of DNA damage checkpoint 1 (MDC1) protein, in which four phosphosites (S1814, S1820, S780, and T1589) are co-regulated with CIT_S440, plays a critical role in DNA repair by functioning as a scaffold protein to recruit various DNA repair proteins (Stewart et al., 2003).

**FIGURE 4:**
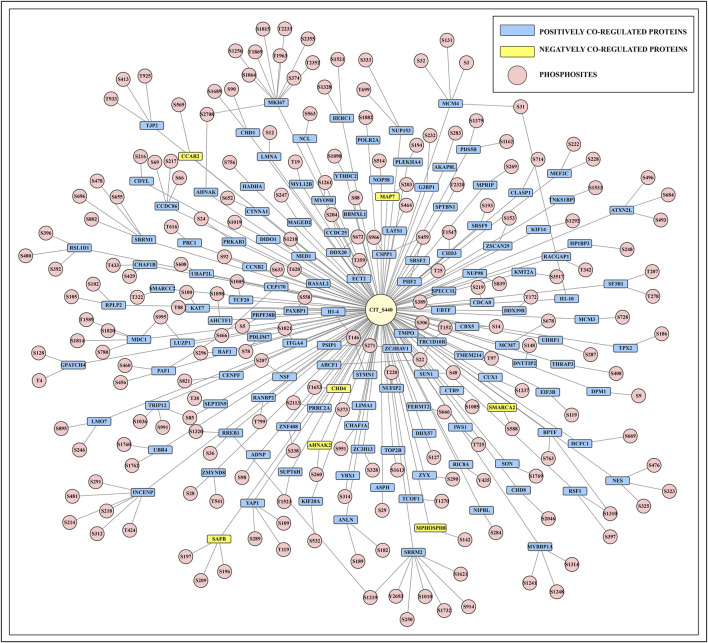
Network diagram illustrating protein-protein interaction centred around CIT_S440. Proteins are represented as nodes.

Similarly, the inner centromere protein (INCENP), which is vital for controlling the Chromosome Passenger Complex (CPC), has six sites (S214, S218, S291, S312, S481, and T424) co-regulated with S440. CIT activity is required for the proper localization of the CPC, and it activates Aurora B, a component of the CPC at the midbody, by phosphorylating the INCENP TSS motif (Samejima et al., 2015; McKenzie et al., 2016). In tissue samples, the proliferation marker protein Ki-67 (MKI67) contributes to cell division and serves as a biomarker for identifying dividing cells. The protein MKI67 has eight sites (S1256, S1815, S1864, S2355, S2708, S374, T1869, and T235) that are co-differentially regulated with CIT_S440 (Miller et al., 2018). The phosphosites of currently known binary interactors that are positively and negatively co-regulated in expression with CIT_S440 are provided in Figure 4.

### 3.6 Potential kinases that can phosphorylate CIT S440

Currently, no experimentally validated kinases are known to phosphorylate the predominant phosphosites of CIT. However, our data identified CDK2_T14 as a highly positively co-regulated upstream kinase of CIT_S440, as predicted by Johnson et al. (2023) (Supplementary Table S1H). Cyclin-dependent kinase 2 (CDK2) is involved in various cellular processes, such as cell cycle regulation, DNA damage, and DNA repair (Liu et al., 2020). Although CDK2 exhibits inhibited enzymatic activity, it shows positive co-regulation with CIT_S440, suggesting a potential functional association (Bártová et al., 2005).

### 3.7 Is CIT_S440 an autophosphorylation site?

Since we have already established the predominant phosphosite of CIT, our focus was to explore whether S440 is an autophosphorylation site. In 2019, a study led by Sugiyama et al. suggested that CIT can auto-phosphorylate itself at S440 (Sugiyamaet al., 2019). Despite previous reports of this autophosphorylation event, we sought to explore its significance by observing motif similarities in the predicted substrates. Upon further analysis of CIT_S440 substrates predicted by Johnson et al., we examined the sequence window (±7) surrounding the phosphosites and identified a conserved motif, “DS/TP” or “PS/TD”, present in 43 proteins, including BUD13, ANP32A, and CENPC (Supplementary Table S1I).

To our interest, we noted that CIT S440 carried a similar phosphomotif, which satisfies the substrate sequence specificity of CIT (“DS/TP” or “PS/TD”). This evidence highlights that S440 could be an autophosphorylation site. BUD13, a predicted substrate for CIT_S440, contains four sites (BUD13_S197, BUD13_S222, BUD13_S271, and BUD13_T159) that exhibit similar motifs, suggesting that CIT may recognize this motif and contribute to its phosphorylation. However, no direct experimental evidence currently confirms that CIT_S440 is an autophosphorylation site or explores the site-specific functions.

We also explored motifs present in the phospho-enriched sequences from a study conducted by Rawar et al., in 2023 and identified the MDC1_S1820 protein phosphosite with a similar motif (Rawat et al., 2023). Based on the FET analysis and the motif analysis of the substrates, we predict CIT_S440 as a potential autophosphorylation site with functional significance to cell cycle regulation.

### 3.8 Identification of downstream substrates that are co-regulated with CIT_S440

To further assess Ser440 as its potential autophosphorylation site and understand the phospho-regulatory pathways of CIT_S440, we analysed the co-regulatory potential of its predicted substrates. Among the 5219 predicted substrates of CIT kinase, the current analysis identified 82 phosphosites in 73 substrates of CIT to be positively co-regulated with the predominant site Ser440, giving an added layer of confidence to these proteins as substrates of CIT (Supplementary Table S1J). Among these, 72 proteins were predicted by Johnson et al., while 10 were predicted by AKID and NetworkIN tools (Johnson et al., 2023; Linding et al., 2008; Parca et al., 2019). Similarly, two substrates predicted by Johnson et al. were identified among the negatively co-regulated high-confidence PsOPs.

For instance, BRIP1_S990, a predicted downstream substrate that is positively co-regulated with CIT_S440, shows a frequency of 12. Notably, this phosphosite has been reported to play a role in the DNA damage response through its interaction with BRCA1 (Zou et al., 2013; Katheeja et al., 2023). Additionally among the 82 substrates, CCDC86 (S66 and S217), DCUN1D4 (S7 and S9), and FRYL (S1978 and T1959) were reported with multiple phosphosites, and a detailed investigation is required to uncover their regulatory functions. However, based on the observations regarding the number of substrates that co-regulated with CIT, we predict Ser440 as a phosphosite associated with CIT kinase activity.

**FIGURE 5:**
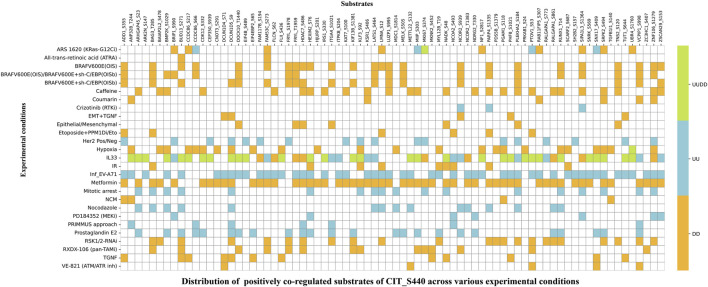
Heat map representing differential expression patterns across multiple experimental conditions. The substrate phosphosites predicted by Johnson et al., which show expression co-regulation with CIT_S440, are represented.

Subsequently, we also analysed the experimentally validated substrates for CIT, MYL9 (S20 and T19) (Yamashiro et al., 2003), and GLI2 (Xing et al., 2014). The GLI2_S149 site was reported to be positively co-regulated with CIT_S440 in a previous study, where mitotic arrest was one of the experimental conditions used in the HeLa cell line. This further validates our hypothesis that CIT_S440 significantly contributes to cell cycle regulation. Since the phosphosites in these substrates did not meet the high-confidence cutoff to be co-regulated with Ser440, we looked into known substrates such as INCENP (which met the high-confidence criteria). In 2016, McKenzie et al. reported INCENP as a substrate of CIT, where CIT phosphorylates at the 783–918 region of INCENP. Our analysis identified six phosphosites corresponding to INCENP with high confidence, such as S214, S218, S291, S312, S481, and T424. Figure 5 illustrates the substrates of CIT_S440 and their differential regulation across different datasets.

## 4 DISCUSSION

CIT is a critical protein with significant clinical relevance, particularly due to its overexpression in cancers such as bladder, breast, and colon, where it drives aggressive tumor phenotypes and poor prognosis. In bladder cancer, CIT is a potential biomarker and therapeutic target, as its overexpression is associated with advanced stages and aggressive phenotypes (Shou et al., 2020). In breast cancer, CIT is associated with tumor growth and invasiveness, while its suppression triggers cell death (Meng et al., 2019; Shou et al., 2020). In colon cancer, CIT promotes tumor growth *via* the p53 pathway, and its silencing reduces proliferation (Wu et al., 2017). However, a study by McKenzie et al. suggests that CIT overexpression alone is insufficient to induce oncogenicity, indicating that additional molecular factors are required for malignant transformation (McKenzie and D’Avino, 2016). These findings highlight the role of CIT as a promising biomarker and therapeutic target node across multiple cancers.

Though protein level information on CIT is known to some extent, the biological significance of CIT phosphorylation is currently unknown. Thus, in the present study, we aimed to derive the phosphoregulatory network of CIT by analysing global phosphoproteomic datasets. We analyzed 758 profiling datasets and 192 differential datasets to explore the phosphoregulatory network of CIT. Our analysis identified CIT_S440 as the predominant phosphorylation site. Subsequent co-regulation analysis revealed 793 PsOPs exhibiting positive co-regulation and 43 PsOPs showing negative co-regulation with CIT_S440. Notably, RSL1D1_S392 and MTA1_S57 displayed positive co-regulation, while RANBP3_S353 and GIT1_S362 showed negative co-regulation. These proteins are associated with key cellular functions. Ribosomal L1 domain-containing protein 1 (RSL1D1) regulates senescence, proliferation, apoptosis, and stress responses (Jiang et al., 2022). Metastasis-associated protein (MTA1) modulates the mitotic spindle checkpoint for proper chromosome segregation (Liu et al., 2023). Ran-binding protein 3 (RANBP3) regulates melanoma cell proliferation and also acts as a cofactor for chromosome region maintenance 1 (CRM1)-mediated nuclear export (Pathria et al., 2016). G protein-coupled receptor kinase-interactor 1 (GIT1) governs vesicle transport, cell adhesion, and cytoskeletal dynamics (Manabe et al., 2002). These associations underscore the functional significance of CIT_S440 in cellular regulation.

To investigate upstream regulation, we identified CDK2 as an upstream kinase primarily associated with cell cycle regulation. Beyond its established role in cell cycle progression, CIT is implicated in DNA damage responses, as CIT-deficient cells exhibit increased double-strand breaks and DNA damage accumulation (Bianchi et al., 2017). However, the molecular mechanism associated with it is currently unknown. Our analysis suggests that CIT has a phospho-regulatory role in influencing DNA repair pathways by phosphorylating its high-confidence substrate BRIP1 at S990. BRIP1 interacts with BRCA1 to promote DNA repair. The phosphorylation of BRIP1 at S990 enables its association with BRCA1, promoting DNA repair (Katheeja et al., 2023; Zou et al., 2013). This work highlights the role of CIT in CIT-BRIP1-BRCA1 signalling to regulate DNA repair mechanisms.

Gene enrichment analysis of binary interactors using DAVID (Sherman et al., 2022) highlighted their involvement in key biological processes, including mitotic cytokinesis, chromatin remodeling, neural development, and DNA repair. Notably, proteins such as NIPBL, TRIP12, MDC1, CHAF1B, UHRF1, and ZMYND8 are enriched in DNA repair pathways. Nipped-B-like protein (NIPBL) serves as a cohesin loading factor critical for DNA repair (Oka et al., 2011). Thyroid receptor-interacting protein 12 (TRIP12) maintains the stability of DNA repair proteins (Inanc et al., 2024). The mediator of DNA damage checkpoint protein 1 (MDC1) mediates DNA damage checkpoint signaling and double-strand break repair (Stewart et al., 2003). Chromatin assembly factor 1 subunit B (CHAF1B) contributes to DNA replication and repair as a chromatin assembly component (Coster and Goldberg, 2010). Ubiquitin-like PHD and RING finger domain-containing protein 1 (UHRF1) acts as a DNA damage sensor, recruiting repair machinery (Tian et al., 2015). Zinc finger MYND domain-containing protein 8 (ZMYND8), a chromatin reader, recognizes histone modifications at damaged sites to facilitate homologous recombination *via* NuRD complex interactions (Carney et al., 2023). The positive co-regulation of these proteins with CIT_S440 suggests involvement of CIT in DNA repair, warranting further experimental validation.

Involvement of CIT in key cellular processes, including cell cycle regulation, cytokinesis, and DNA repair, highlights its multifaceted impact on cellular processes. Specifically, the phospho-regulatory network of CIT_S440, a predicted autophosphorylation site, emerges as a key regulator, with hyperphosphorylation observed across multiple cancer types. This positions CIT_S440 as a promising onco-phosphosite, warranting future studies to focus on elucidating molecular mechanisms through which CIT_S440 contributes to cancer progression. Nevertheless, we acknowledge that although the present study is based on experimentally derived phosphoproteomics datasets, contextual evaluation of the role of CIT phosphosites warrants targeted experimental validation.

### 4.1 Limitations of the study

We acknowledge that the present study does not involve reprocessing of raw data and hence correlative nature of the network analysis be considered under the premises of limitations arising from the heterogeneity of the publicly available phosphoproteomics datasets derived from diverse experimental conditions, instrumentation, peptide assignment pipelines, and normalisation methods. To minimise the discrepancies in the identification of high confident co-phosphoregulatory sites, we applied stringent inclusion/exclusion criteria along with FET analysis. The global approach undertaken in this study engages the enrichment and analysis of phosphosites in CIT based on phosphoproteins (including interactors) co-regulated with it. This study can be used as a platform for the evaluation of the filtered phosphosites for the assessment of their functional roles based on targeted experimental approaches.

## 5 Conclusion

Our study extracted the first phospho-regulatory network of CIT kinase, with a primary focus on its predominant phosphorylation site, Ser440, which is also proposed to be associated with kinase activity. CIT_S440 was the most frequent phosphosite identified in 758 profiles and 192 differential datasets. Through bioinformatic analysis and statistical evaluation, we identified protein phosphosites that were highly co-regulated with CIT_S440. The phosphorylation site Ser440 modulates the role of CIT in DNA repair and cell cycle regulation, with the functionally related proteins positively co-regulated with Ser440. With hyperphosphorylation of CIT_S440 observed in multiple cancers such as breast cancer, colon cancer, and bladder cancer, Ser440 could be a significant onco-phosphosite that is central to several cellular checkpoint signaling. Our study provides the first comprehensive phosphosite-centric signaling landscape of CIT_S440, which can serve potential implications for future therapeutic strategies.

## Data Availability

The original contributions presented in the study are included in the article/Supplementary Material, further inquiries can be directed to the corresponding author.

## Glossory

CIT: Citron Rho-interacting serine/threonine kinase
RMLC: phosphorylates Regulatory Myosin Light Chain
HR: Homologous recombination
PsOPs: phosphosites in other proteins
FET: Fisher exact test
UDDU: Negatively co-differentially regulated
UUDD: Positively co-differentially regulated
SRRM2: Serine/arginine repetitive matrix 2
MDC1: The Mediator of DNA damage checkpoint 1
INCENP: Inner Centromere Protein
MKI67: Proliferation marker protein Ki-67
NIPBL: Nipped-B-like protein
TRIP12: Thyroid receptor-interacting protein 12
CHAF1B: Chromatin assembly factor 1 subunit B
UHRF1: Ubiquitin-like PHD and RING finger domain-containing protein 1
ZMYND8: Zinc finger MYND domain-containing protein 8
DAVID: The Database for Annotation, Visualization and Integrated Discovery
TOPBP1: DNA topoisomerase 2-binding protein 1
BRIP1: BRCA1-interacting protein 1
RPA1: Replication protein A 70 kDa DNA-binding subunit
RSL1D1: Ribosomal L1 domain-containing protein 1
MTA1: Metastasis-associated protein MTA1
RANBP3: Ran-binding protein 3
GIT1: G protein-coupled receptor kinase-interactor 1 GIT1
BRIP1: BRCA1-interacting protein 1 BRIP1
RAD18: E3 ubiquitin-protein ligase RAD18
RECQL: ATP-dependent DNA helicase Q1 RECQL
KIF20A: Kinesin Family Member 20A KIF20A
CDCA8: Cell division cycle-associated protein 8 CDCA8
RBD: Rho/Rac-binding domain
PH: pleckstrin homology
CNH: Citron-Nik1 homology
